# Supplementary material for: The incidence of postoperative periprosthetic femoral fracture following total hip replacement: An analysis of UK National Joint Registry and Hospital Episodes statistics data
Source: PLoS Med. 2024 Oct 1;21(10):e1004462. doi: 10.1371/journal.pmed.1004462 (PMC11444412; doi:10.1371/journal.pmed.1004462)
Supplement: S2 Table — (DOCX) [file pmed.1004462.s002.docx]

**Supplemental table 2 - Breakdown of reasons for revision including items contained within "Other"**

| **Indication for revision** | **N** |
| --- | --- |
| Dislocation/subluxation | 4060 |
| Infection | 3527 |
| Periprosthetic fracture stem | 3186 |
| Aseptic loosening stem | 2567 |
| Aseptic loosening socket | 2422 |
| Pain | 1433 |
| Other indication for revision | 978 |
| Malalignment socket | 977 |
| Wear of acetabular component | 862 |
| Lysis socket | 538 |
| Malalignment stem | 497 |
| Lysis stem | 472 |
| Adverse soft tissue reaction to particle debris | 385 |
| Implant fracture stem | 351 |
| Periprosthetic fracture socket | 316 |
| Dissociation of liner | 302 |
| Implant fracture socket | 259 |
| Implant fracture head | 101 |
| Incorrect sizing head socket mismatch head | 69 |
| Incorrect sizing head socket mismatch socket | 61 |
| Incorrect sizing MDS2 head socket mismatch | 9 |
