# Supplementary material for: The incidence of postoperative periprosthetic femoral fracture following total hip replacement: An analysis of UK National Joint Registry and Hospital Episodes statistics data
Source: PLoS Med. 2024 Oct 1;21(10):e1004462. doi: 10.1371/journal.pmed.1004462 (PMC11444412; doi:10.1371/journal.pmed.1004462)

**Supplemental figure 1 - Histogram depicting length of stay in the acute hospital after identification of post-operative periprosthetic femoral fracture (POPFF)**


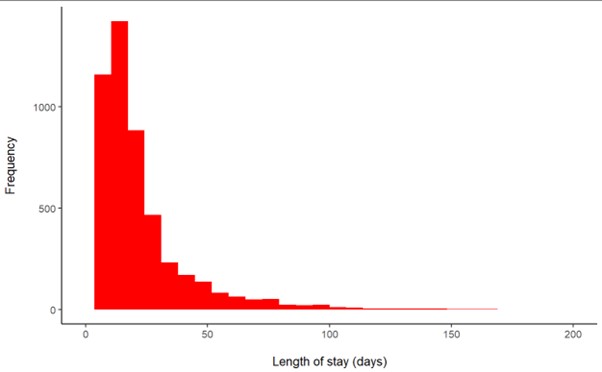

Supplement: S1 Fig — (DOCX) [file pmed.1004462.s003.docx]
