# Supplementary material for: The incidence of postoperative periprosthetic femoral fracture following total hip replacement: An analysis of UK National Joint Registry and Hospital Episodes statistics data
Source: PLoS Med. 2024 Oct 1;21(10):e1004462. doi: 10.1371/journal.pmed.1004462 (PMC11444412; doi:10.1371/journal.pmed.1004462)
Supplement: Attachment — Submitted filename: Response_to_reviewers.docx [file pmed.1004462.s005.docx]

16^th^ May 2024

Dear Ms Sunny,

**PMEDICINE-D-24-01147R1 - Postoperative periprosthetic femoral fracture is the leading cause of reoperation following total hip replacement: An analysis of linked data from the National Joint Registry and Hospital Episodes Statistics.**

We thank you and the reviewers for your helpful reviews and comments on our manuscript. We have addressed your comments and queries as detailed below and have altered the manuscript accordingly. We would be very grateful for your further review for suitability for publication in PLOS Medicine.

Thank you very much again for your time,

Yours sincerely,

Jonathan T Evans on behalf of the authors

Clinical Senior Lecturer in Trauma & Orthopaedics

University of Exeter, UK

**Editorial comments:**

**1)	The editorial team agree that the authors should be commended on undertaking research on an important complication of total hip replacements, and we are grateful for the opportunity to consider your work. However, we do agree with the reviewers’ points with regards to the handling and reporting of data, and also the need to further justify some of the assertions made. All reviewer comments will need to be addressed in full before we can consider the manuscript further.**

Thank you very much for the comment. We have addressed all the comments made in the document below and we would be very grateful for your reconsideration.

***2)	Financial Disclosures***

***Please provide further information about the funding body, including the funder’s name in full. Also, please describe any affiliations or contracts with the National Joint Registry, if applicable.***

We have added the following text in to the finding section at the end of the abstract.

*“This study was funded by Orthopaedic Research United Kingdom (ORUK). MRW is Principal Investigator (PI) of the National Joint Registry lot 2 contract (statistical analysis) team which is hosted by his employer, the University of Bristol; JTE is a member of the lot 2 contract team.”*

***3)	Data Availability***

***Thank you for including a statement regarding data availability. Please also include a statement about accessing Hospital Episode Statistics data.***

I have updated this in the submission portal.

*“HES Admitted Patient Care data was made available via same route under sublicensing agreement between NJR and NHS England”*

***4)	Reporting guidance***

***Please ensure that the study is reported according to the RECORD (STROBE if you feel it is more appropriate) guideline and include the completed RECORD checklist as Supporting Information. Please add the following statement, or similar, to the Methods: "This study is reported as per the Reporting of studies Conducted using Observational Routinely-collected health Data (RECORD) Statement (S1 Checklist)."***

***When completing the checklist, please use section and paragraph numbers, rather than page numbers.***

***The relevant guidance can be found here: https://eur03.safelinks.protection.outlook.com/?url=https%3A%2F%2Fwww.equator-network.org%2Freporting-guidelines%2F&data=05%7C02%7Cj.t.evans%40exeter.ac.uk%7C7c286d1410e5403306db08dc7584fe94%7C912a5d77fb984eeeaf321334d8f04a53%7C0%7C0%7C638514461262600671%7CUnknown%7CTWFpbGZsb3d8eyJWIjoiMC4wLjAwMDAiLCJQIjoiV2luMzIiLCJBTiI6Ik1haWwiLCJXVCI6Mn0%3D%7C0%7C%7C%7C&sdata=zjOBAdUicVMVcbxClXjA7ZISl44NoL6i1wDq3V5E5BM%3D&reserved=0***

Thank you. We have now included the RECORD checklist.

***5)	Statistical reporting***

***Please quantify the main results with 95% CIs and p values.***

***When reporting p values please report as <0.001 and where higher as p=0.002, for example. When reporting 95% CIs please separate upper and lower bounds with commas instead of hyphens as the latter can be confused with reporting of negative values.***

All values given as 95% CI’s checked and commas replacing other methods.

All p values checked and comply with requirements.

***Please include the actual amounts and/or absolute risk(s) of relevant outcomes (including NNT or NNH where appropriate), not just relative risks or correlation coefficients. (example for absolute risks: PMID: 28399126).***

Absolute values have been reported with unadjusted Kaplan Meier estimates of survival.

***Please include any important dependent variables that are adjusted for in the analyses.***

This study does not use statistical adjustment of estimates.

***6)	Prespecified analysis plan/study protocol***

***Did your study have a prospective protocol or analysis plan? Please state this (either way) early in the Methods section.***

This study did not have a prospective protocol or analysis plan published however the proposed methods were submitted to the NJR research sub-committee for approval when applying for data access. We have now added a statement into the methods to highlight this.

*“Approval for the study and the planned methodology was granted by the NJR research sub-committee (RSC2017/20 and RSC2019/07) prior to data access* *and no data driven changes to analyses took place.”*

***a) If a prospective analysis plan (from your funding proposal, IRB or other ethics committee submission, study protocol, or other planning document written before analyzing the data) was used in designing the study, please include the relevant prospectively written document with your revised manuscript as a Supporting Information file to be published alongside your study, and cite it in the Methods section. A legend for this file should be included at the end of your manuscript.***

n/a

***b) If no such document exists, please make sure that the Methods section transparently describes when analyses were planned, and when/why any data-driven changes to analyses took place.***

A statement to this effect has been added in the first paragraph of the methods.

*“Approval for the study and the planned methodology was granted by the NJR research sub-committee (RSC2017/20 and RSC2019/07) prior to data access and no data driven changes to analyses took place.”*

***c) In either case, changes in the analysis-- including those made in response to peer review comments-- should be identified as such in the Methods section of the paper, with rationale.***

***For all observational studies, in the manuscript text, please indicate: (1) the specific hypotheses you intended to test, (2) the analytical methods by which you planned to test them, (3) the analyses you actually performed, and (4) when reported analyses differ from those that were planned, transparent explanations for differences that affect the reliability of the study's results. If a reported analysis was performed based on an interesting but unanticipated pattern in the data, please be clear that the analysis was data-driven.***

As this study was focused on reporting the incidence and epidemiology of post operative periprosthetic fracture there were no specific hypotheses.

***7)	Abstract layout***

***Please structure your abstract using the PLOS Medicine headings (Background, Methods and Findings, Conclusions), i.e. the Methods should be combined with Findings to make one section.***

Thank you for highlighting this, we have amended the abstract to keep it in line with PLOS Medicine guidance.

***8)	Author summary***

***At this stage, we ask that you include a short, non-technical Author Summary of your research to make findings accessible to a wide audience that includes both scientists and non-scientists. The authors summary should consist of 2-3 succinct bullet points under each of the following headings:***

***•	Why Was This Study Done? Authors should reflect on what was known about the topic before the research was published and why the research was needed.***

***•	What Did the Researchers Do and Find? Authors should briefly describe the study design that was used and the study’s major findings. Do include the headline numbers from the study, such as the sample size and key findings.***

***•	What Do These Findings Mean? Authors should reflect on the new knowledge generated by the research and the implications for practice, research, policy, or public health. Authors should also consider how the interpretation of the study’s findings may be affected by the study limitations. In the final bullet point of ‘What Do These Findings Mean?’, please describe the main limitations of the study in non-technical language.***

***The Author Summary should immediately follow the Abstract in your revised manuscript. This text is subject to editorial change and should be distinct from the scientific abstract. Please see our author guidelines for more information: https://eur03.safelinks.protection.outlook.com/?url=https%3A%2F%2Fjournals.plos.org%2Fplosmedicine%2Fs%2Frevising-your-manuscript%23loc-author-summary&data=05%7C02%7Cj.t.evans%40exeter.ac.uk%7C7c286d1410e5403306db08dc7584fe94%7C912a5d77fb984eeeaf321334d8f04a53%7C0%7C0%7C638514461262603771%7CUnknown%7CTWFpbGZsb3d8eyJWIjoiMC4wLjAwMDAiLCJQIjoiV2luMzIiLCJBTiI6Ik1haWwiLCJXVCI6Mn0%3D%7C0%7C%7C%7C&sdata=g4c1jag4zmYWGYQtuUonxsPVH9%2BxpUtQB3FXrEpqFBU%3D&reserved=0***

Thank you very much. We have now included this in the revised manuscript but can also be seen here:

Author summary

*Why was the study done?*

- When the thigh bone supporting a hip replacement breaks because of injury, it is called a periprosthetic fracture of the femur. Patients are usually treated with major surgery, which is associated with significant risk of complications, high cost and even death.
- Currently the best estimates of how often these injuries occur after hip replacement in England are made using data which only counts when fractures are treated with one surgical method, revision and therefore miss a large proportion of cases.
- This study is the first large study combining hospital data and the best currently used source to get a much more complete picture of when these fractures occur. In addition to revision, the study was able to capture the other common treatment method i.e. fixation, thereby providing a comprehensive and more meaningful review.

*What did the researchers do and find?*

- A very large data set of all hip replacements performed in England were matched to health data on hospital admissions to find patients who were treated with either exchange of implants (revision) and fixation of the fracture without exchange of implants.
- This study found that previous estimates of periprosthetic fractures only represented about half of the actual number treated in hospitals over the past two decades. When cases treated with revision and fixation are combined, periprosthetic fracture is the most common cause of major re-operation after hip replacement.
- Patients who go on to sustain a fracture tended to be older and frailer at the time of the first hip replacement than those who don’t go on to break. One in twenty-five patients with fractures died within 30 days of their operation, and the hospital length of stay was over two weeks.

*What do the research findings mean?*

- These results demonstrate that periprosthetic fracture after hip replacement is the biggest problem facing patients with a hip replacement and the scale of the problem has previously been underestimated.
- Health care providers and researchers should prioritise the prevention and care for these vulnerable patients.

***9)	Introduction layout***

***Please address past research and explain the need for and potential importance of your study. Indicate whether your study is novel and how you determined that. If there has been a systematic review of the evidence related to your study (or you have conducted one), please refer to and reference that review and indicate whether it supports the need for your study.***

Thank you, we feel the introduction now achieves this. We have also shortened it considering comments from other reviewers.

***10)	Discussion layout***

***Please present and organize the Discussion as follows: a short, clear summary of the article's findings; what the study adds to existing research and where and why the results may differ from previous research; strengths and limitations of the study; implications and next steps for research, clinical practice, and/or public policy; one-paragraph conclusion.***

Thank you. We have edited the discussion considerably in line with reviewer’s comments and hope it reaches your standards now.

***Reviewer #1:***

***Realising that NJR data only captures revision procedures, this study linked data from the NJR to Hospital Episode Statistics to also detect re-operations for post-operative periprosthetic fracture that were treated by fracture fixation without revision. They found over half of re-operations were fracture fixations and therefore not included in the NJR. Further analysis of patient demographics, timing of fracture and cost of treatment is included.***

***The messages from this study are important and highlight a number of concerns about POPFF, however the paper has a number of areas in which it could be improved.***

***The major problem is that the authors state (in multiple places in the paper) that this study is of the "true" incidence of POPFF, but it is not, as they also point out that some POPFF are treated non-operatively and these are not included. This paper therefore studies only re-operation for POPFF, and this needs to be made clear in the manuscript.***

Thank you for this comment. The reviewer is absolutely correct, and we agree that we were not explicit about this, the manuscript has been revised to state that results represent an attempt to identify incidence of *surgically treated* POPFF. We have removed any reference to the word “true” in this context.

We have also specified in our aim section that we are only looking at patients who have a surgically managed POPFF.

*“The aim of this study was to identify reoperation for POPFF after primary total hip replacement using a linked implant registry and healthcare dataset. This will allow us to investigate the incidence, changes in incidence, demographics of patients sustaining surgically managed POPFF and outcomes after fracture including length of stay and mortality.”*

***A second major problem is that re-operation for POPFF is compared to only revision for other causes (such as dislocation and infection), so the claim that POPFF is the major cause of re-operation may be incorrect. While there is some discussion about this point, the comparison is unbalanced, and should be better qualified or removed. This consideration also needs to be applied to comparisons of demographic groups and mortality.***

Thank you for highlighting this. It is an area that we have had much internal discussion over. We have amended the text (see below) and hope that it is now more balanced. Whilst we appreciate that there are many causes of major reoperation for a failing hip replacement, we do not believe that other causes of reoperation which are also likely missed in the NJR, such as closed reduction of a dislocation or surgical management of infection (without changing of components) require the same amount of surgical time and resources and are associated with the same blood loss, morbidity and complications. If an initial DAIR procedure is unsuccessful, the likelihood is that the patient will then proceed to a revision which will be captured in the registry. The reviewer is correct in the assertion that the vast majority of dislocations that undergo closed reduction will be missed, but considering that only 11% of patients with a dislocation go on to have a revision (<https://pubmed.ncbi.nlm.nih.gov/38276104/>) and the majority of these will be captured by the registry, we do not feel it to be equivalent to the POPFF. Other causes of major reoperation because of failure of a hip replacement are likely to be recorded in the NJR. We feel that POPFF represents the most frequently encountered cause of major reoperation despite other causes.

We have added a clarification of this argument in the `Strengths and weaknesses` paragraph at the end of the discussion.

*“We have successfully identified POPFF treated with internal fixation and added them to the already known revisions within the NJR but have not addressed the same issue for two other key reasons for failure infection and dislocation. Dislocated THRs treated with closed or open reduction without modification or changing of the implants will not be identified in this cohort, this is hard to assess using admitted patient HES data (as in this study) as many dislocations are treated with closed reduction in the Emergency Department and may not be admitted to hospital which will not be captured in the available linked data. Recent evidence has demonstrated that the cumulative incidence of dislocation may be as high as 0.9% within 30 days but as few as 11% of these patients undergo revision surgery for dislocation.18 In a similar manner, infected THRs treated with a debridement and no exchange of implants were not captured prior to June 2018. Operations for infection including debridement and implant exchange will have been captured in the NJR dataset. Reoperation for infection is associated with large volume blood loss.19 Addition of operations for dislocation and infection will improve the validity of large registry datasets in the future and allow for accurate planning and prioritisation of resource allocation and focus for future research.”*

***Other criticisms include:***

***Introduction:***

***Common use descriptions (such as thigh bone, re-do surgery) should be removed as this is a scientific manuscript not a lay article.***

Thank you for your comments. The manuscript has been edited accordingly and we have restricted the use of these terms to the lay language sections.

***The aims of the study need to be more clearly stated.***

The aims of the study have been clarified:

*“The aim of this study was to identify reoperation for POPFF after primary total hip replacement using a linked implant registry and healthcare dataset. This will allow us to investigate the incidence, changes in incidence, demographics of patients sustaining POPFF treated surgically and outcomes after this treatment including length of stay and mortality.”*

***Methods:***

***The inclusion/exclusion criteria need to be clearly stated in one place. Some of this information is in the first paragraph and some in the second.***

Thank you for highlighting this, the inclusion criteria have now been specified more accurately and all in one place.

*“Patients were included if they had undergone primary THR between 1 January 2004 and 31 December 2020 and had undergone a first reoperation for POPFF after the day of primary surgery.”*

***How incidence of POPFF is calculated needs to be stated.***

A description has been added to the methods.

*“Incidence was estimated using a count of new cases per year or prosthesis time incidence rate (PTIR), which was number of new events per 1000 years patient time with a hip replacement.”*

***It is unclear what sensitivity analyses were carried out.***

We did not carry out any sensitivity analyses. We performed analyses restricted by age and indication for primary operation but wanted to keep the message broad at this stage considering it is an epidemiological paper.

***There is no mention of how hospital LOS data were obtained or information on hospital costs.***

The methods have been updated as follows.

*“Length of stay was estimated as the number of whole days between date and time of admission and time of discharge from hospital. Inpatient stay costs were estimated using an estimate of £586.59 per bed day^16^ .”*

***The patient/public involvement would be better placed toward the end in the acknowledgement section.***

We have added a statement to this effect in the acknowledgement section.

***Results:***

***In this section the results should be clearly stated and interpretation left for the discussion. For example, comments about previous studies using NJR data, or bimodal distribution of fractures.***

Statements relating to the interpretation of results has been moved to discussion points or deleted.

***Figure 2 is the "reasons for revision" not "failure", as some revisions are carried out with a well-functioning prosthesis, such as for infection. Also what is the difference between the 7th reason for revision "other indication for revision" and the last reason "other"? This figure "shows" the frequency of each reason for revision, the reader "visualises". Part 2 of this should be removed for the reason above.***

Thank you. Failure has been changed to revision as per your feedback.

“Other indication for revision” is a pre-existing category supplied in the original data. “Other” is an added grouping of rare indications, which has been used by us to make the chart more easily readable. These categories have been left separate to preserve as much of the original data structure as possible.

***Similarly, the word "failure" should be removed from the total incidence rate per 1000 patient years. These are re-operations not failures. This is also a mis-leading calculation as it does not include all re-operations.***

Thank you, we have removed the word failure and replaced it with reoperation (for revision or fixation).

*“giving an incidence rate of 4.09 (95% CI 4.03, 4.14) reoperations (for revision or fixation) per 1000 prostheses years”*

***Figure 3 shows the number of POPFF by year, not the incidence as stated in the text.***

Thank you, changes have been implemented.

*“The number of new cases of POPFF amongst patients who had a primary hip replacement recorded in the NJR increased each year (Figure 3).”*

***The term PTIR needs definition.***

Thank you for highlighting this. We have made this change.

*“Incidence was estimated using a count of new cases per year or prosthesis time incidence rate (PTIR), which was number of new events per 1000 years that a prosthesis has cumulatively been in place.”*

***I do not understand how the calculated overall incidence of revision and fracture fixation per 1000 patient years is the same as for those over 70 at time of THR (4.09 for both). I would expect these to be different. A comparison of those 70 and over with those less than 70 would be helpful.***

Thank you, this may be because the risk of revision is greater in younger patients versus older patients and there is a significant non-informative competing risk of death leading to censorship in patients older than 70. As you can see from the results there was a large increase in POPFF reoperation between all patients and those above 70 (0.92 to 1.31). Whilst younger patients tend to experience more reoperation risk, a smaller proportion of their overall events are POPFF reoperations. This has not been fully explored because it was beyond the scope of the aim of the study but is well worth future investigation.

***Comparison by primary diagnosis may be clearer if grouped into OA, Fractured NOF and Other.***

Thank you for the suggestion. As you are aware most patients undergo THR for OA of the hip in this setting with over 90% of cases in the NJR having this recorded as an indication for primary THR, so the result for all patients is analogous to this group. We have grouped according to over 70, non-OA diagnoses and NOF because these are key groups where the risk of POPFF and overall reoperation appears to increase. We feel that this is an important part of the message to policy makers and surgeons, when planning services and treatments in the future.

***The timing of fracture treatment section needs to simply state the result.***

Thank you, discussion point deleted.

***Figure 4 should be titled the cumulative incidence of POPFF.***

Thank you, changes have been implemented.

***The display of mortality after fracture treatment may be more easily interpreted if shown as survival. Additionally, a comparison of survival after fracture treatment to survival after revision for other causes in figure 5 would be helpful.***

Thank you, we have chosen proportion of patients who have died using cumulative incidence because it focuses the reader on patient mortality, rather than survival, which is a key outcome measure according to our patient participation group who support us in this work. We feel it is also better to present different data in a paper in a similar format to aid interpretation, as has been done here with the cumulative incidence of postoperative periprosthetic femoral fracture and the cumulative incidence of mortality. In addition, cumulative incidence plots are preferred because they have a reduced plot size. For clarity we have edited the title to make this clearer.

***The calculation of cost is difficult to follow without knowing the mean LOS. If in 2015 for instance the number of POPFFs was 410 (as shown graphically), and the median LOS is used, the result is far short of 10,000 hospital bed days. The LOS needs to be at the upper confidence level of 26 to achieve this.***

Unfortunately, the data is very skewed as expected and a mean is not appropriate, as highlighted here, when applied to highly skewed data, it leads to erroneous estimates. There were many unfortunate patients with incredibly long lengths of stay which skewed the result significantly.

The methods have been updated to explain the calculation of cost estimates.

*“Length of stay was estimated as the number of whole days between date and time of admission and time of discharge from hospital. Inpatient stay costs were estimated by multiplying total inpatient bed days by an estimated cost of £586.59 per bed day”*

***Discussion:***

***The statement about POPFF being the greatest reason for re-operation needs to be qualified.***

Thank you, please see edit:

*“We have observed that in our study population, post-operative periprosthetic femoral fracture is the most common indication for major reoperation following a total hip replacement when revision and fixation operations are captured.”*

***I don't understand how frequency, mortality and cost results make this group "vulnerable".***

Our data show that patients who experience POPFF are older and frailer, accepted markers of vulnerability, than those that do not. This is then reflected in outcomes such as higher mortality and associated costs related to increased length of stay. The paragraph has been edited:

*“Patients with POPFF were older and frailer than patients without POPFF and 90-day mortality for patients who have undergone revision or fixation of a POPFF was more than five times greater than for any other type of revision operation for hip replacement.^5^ The cost of looking after patients with these injuries (in bed days alone) may be over £5 million a year without accounting for theatre time, equipment and follow up/subsequent complications. These results emphasise the significant vulnerability of this patient group in relation to other groups of patients with problematic hip replacement.”*

***In the discussion there should be mention that only 80% of revisions for POPFF were captured in the NJR, and the implications of this.***

Thank you, we have used diverse data sources to try and reduce inaccuracies in any single data source. Our paper relies on the validity of OPCS and ICD coding which, unlike NJR data is not subject to data audit. We cannot assume that any of the data is perfect and hence why we have tried to mitigate this with information from multiple sources.

We have added comment on this:

Principal findings:

*“As implant registers turn from implant monitoring tools to research tools, efforts should be made to increase the clinically important data captured so that results are reflective of the real-world patient experience.”*

Strengths:

*“In this study information form hospital coding data has been used to identify a greater number of revision operations, which may have previously been missed. These methods are likely to improve accuracy of the data and subsequent observations.”*

Future work:

*“Implant survival estimates from the NJR may change once these additional reoperations are included as an endpoint of implant survival, and this effect should be explored in further work”*

***The discussion should include the timing of the fracture and how it differs from previous presumptions.***

Fracture timing is likely to be an implant dependent phenomenon. e.g. cementless stem fractures tend to occur in the period prior to osseointegration, cemented stems occur more steadily over time. Other work (under review) is currently addressing this point and we feel that this current piece of work is meant to emphasise the more general aspects of POPFF to a wider audience, without an implant focus.

***The discussion could include mention of the patient impact.***

Patient impact is difficult to accurately describe in this study given the limited patient level data (e.g. PROMS, qualitative data etc). We have referred only to Length of stay and delay to surgery as a very superficial measure of this. We agree that this is an important issue and We have added a suggestion for this to form the basis of future research:

*“The effect of POPFF on the patient experience in the short and longer term requires further investigation.”*

***I don't understand how patients with POPFF are "underserved". They all would have been adequately treated.***

Thank you. This has been clarified.

*“Previous failings in the identification of patients who have suffered periprosthetic fracture mean that this population has not been prioritised appropriately, or the requirements for designing adequately resourced services to appropriately meet their needs may have been underestimated. Given the relative scale of the problem, focused efforts should be made to tackle POPFF with adaptation of surgical training, health care pathways, and funding for care and research.”*

***Implant survival estimates in the NJR will not change by adding fracture fixation data, as the implant will still be un-revised.***

This depends on what the chosen end point is. The femur is fractured by forces imparted on it through the femoral stem. The risk of POPFF varies significantly between stem designs and the fracture event is in part, the result of the stem in the femur at the time of fracture. Whether or not the stem can be retained in the subsequent treatment is not really of interest to the patient or many surgeons and we feel that the need for fixation is an important outcome given it is still major surgery with substantial risk and morbidity. We have clarified this point in the text.

***The discussion lacks detail of how this study compares to previous studies looking at POPFF with only one mentioned.***

We have updated the discussion to include reference to other revision only studies, where the prevalence was up to 50% of the reported prevalence in this study.

*“We estimate that the overall prevalence of periprosthetic fracture was 0.6% which was at least double that of previous estimates from large national registries.”*

***It may be helpful to the reader to have a comparison of the groups treated by revision +/- fixation to those treated by fixation alone.***

We agree that this would be an interesting topic to address but unfortunately, we do not feel that is within the current scope and length limitations for this study. We have added this as a suggestion for further research in the discussion.

*“The effect of POPFF on the patient experience in the short and longer term requires further investigation, with particular reference to the specific outcomes of fixation and revision as treatment methods.”*

***There is no discussion of prosthesis characteristics for those treated for a POPFF.***

Thank you, this is an important area for research and is the focus of a follow-on paper from this study. We felt that there was too much information for inclusion in one single paper. We also felt that the epidemiological information from this study was more suited for a general interest journal and the prosthesis specific work was more suited for a specialty journal.

***Reviewer #2:***

***Thank you for opportunity to review this interesting paper. I think that the main findings highlight the previously under-estimated burden of periprosthetic fractures. However, in my opinion, the manuscript need some reconstruction. I would also prefer line numbers in the manuscript to make the communication easier.***

***Specific comments:***

***Abstract:***

***Please report confidence intervals for incidences.***

Thank you, confidence intervals have been stated for all incidence rates throughout the paper.

***Introduction:***

***In general I would prefer shorter introduction. Some parts may be considered to shortened or moved to the discussion. Some previous epidemiology of the periprosthetic femoral fractures should be included, including the estimated incidence and predisposing factors.***

Thank you, the introduction has been, shortened and reference has been made to incidence and risk factors for POPFF.

***“In 2019, the National Hip Fracture Database (NHFD) started collecting information on POPFF but these data are crude, do not include implant information and do not yet appear to be universally completed.1" Please define "universally complete"? What is missing?***

Thank you, this statement has been deleted as part of the shortening of the introduction in response to previous comments.

***If you cite Bottle et al., please shortly repeat their main findings, not only some part of discussion and weaknesses of their study.***

Thank you, the main findings have been included briefly in the introduction.

***Methods***

***Please define all outcomes you are reporting in the results. Now the results section is very heterogeneous and does not match the outcomes defined. For example, some calculation of the costs are reported in the results but not once mentioned in the methods.***

Thank you for this comment, the methods section has been comprehensively rewritten to clarify all primary and secondary outcomes with explanation of the methods involved in each.

***Statistical analysis-paragraph includes mostly information that should not be under statistics-section in my opinion. Please restructure the methods section and leave only part that consider the statistical analyses here.***

Thank you for this comment, the methods section has been comprehensively rewritten to clarify the statistical analysis paragraph.

***Results***

***Figure 2 highlights the missing POPFFs in the NJR data. However, in the limitations of the study you mention that THA infections treated with debridement without change of components are not recorded in the data. Therefore, if I have understood correctly, this figure gives false comparison of the burden of POPFFs compared with infections. Also, the dislocations treated with closed reposition are missing. I find this figure misleading and would consider removing or altering it. If you decide to keep this figure, this problem should be clearly mentioned in the discussion.***

Thank you for the comment, the title of figure two has been edited to make the comparison clear. This is a discussion point raised by another reviewer and we have added a section in to the discussion to address this. For the purposes of this study, we feel that the figure is a useful representation of the effect of including POPFF and detail regarding the issue of missed dislocation and infection operations has been added to the discussion section including a short discussion on missing cases to treat dislocation and infection.

*“We have successfully identified POPFF treated with internal fixation and added them to the already known revisions within the NJR but have not addressed the same issue for two other key reasons for failure infection and dislocation. Dislocated THRs treated with closed or open reduction without modification or changing of the implants will not be identified in this cohort, this is hard to assess using admitted patient HES data (as in this study) as many dislocations are treated with closed reduction in the Emergency Department and may not be admitted to hospital which will not be captured in the available linked data. Recent evidence has demonstrated that the cumulative incidence of dislocation may be as high as 0.9% within 30 days but as few as 11% of these patients undergo revision surgery for dislocation.^18^ In a similar manner, infected THRs treated with a debridement and no exchange of implants were not captured prior to June 2018. Operations for infection including debridement and implant exchange will have been captured in the NJR dataset. Reoperation for infection is associated with large volume blood loss.^19^ Addition of operations for dislocation and infection will improve the validity of large registry datasets in the future and allow for accurate planning and prioritisation of resource allocation and focus for future research.”*

***"The incidence of POPFF amongst patients who had a primary hip replacement recorded in the NJR appears to be rising by year (Figure 3). This figure must be interpreted with caution however as the early part of the chart represents a time where not as many hips had been input into the NJR and were therefore at risk of POPFF"***

***This type of speculation inn valid, however it should be in discussion, not in results.***

Speculation has been removed from the results.

***"It is commonly considered that POPFF occur early after implantation (and may represent a missed intraoperative fracture) or that they occur late after implantation (e.g. 10 years or more) secondary to loosening and/or lysis.15" Never use references in the results section, again, this to the introduction or to discussion.***

Thank you, this has been removed.

***Demographics: Start the result section with demographics and give it short.***

Thank you for this suggestion. We have taken this on and moved the demographics section earlier within the results.

***Mortality: The mortality rate is known to be high in periprosthetic fractures. Could you compare the mortality rates between patient who suffered a POPFF and those who did not?***

We have considered this, but we have chosen to compare mortality between those undergoing revision for non POPFF causes as a comparison. We feel that this is a more valid approach and gives a sense of the effect of the fracture and surgery against that experienced by other patients with problematic hip replacement. We hope that this highlights the relative difficulties these patients face.

***Discussion***

***The first paragraph should focus on the main finding of this study. The following part of the first paragraph could be moved to later discussion and partly added in the conclusions: "A non-revision reoperation data collection form was added to the NJR in June 2023 but the uptake of this and capture rate is not yet known. 90-day mortality for patients who have undergone revision or fixation of a POPFF was greater than five times more than for any other type of revision operation for failing hip replacement.5 The cost of looking after patients with these injuries (in bed days alone) may be over £5 million a year without accounting for theatre time, equipment and follow up/subsequent complications. These results emphasise the significant vulnerability of this patient group in relation to other groups of patients with problematic hip replacement."***

Thank you, the paragraph has been edited and moved to a later part of the discussion dealing with future implications of this work.

***Please compare your results in previous studies. What has been the estimated incidence of POPFFs inn the previous studies?***

Thank you this has been added to the introduction and comparison in the discussion.

***Conclusions: The economic burden could be mentioned here, but not in the principal findings of the study since this was not a cost-analysis study.***

Thank you this has been removed.

***Reviewer #3:***

***Using data from the English(?) National Joint Registry and the National Health Service, the incidence of post-operative periprosthetic femoral fracture after total hip replacement from 2004-2020 inclusive was estimated.***

***1. Please state what country's national joint registry is being used in the title and in the Background section of the Abstract.***

Thank you for asking for clarification on this. This is a common problem as the NJR does not include Scotland but does include all of the other home nations. As a result, we cannot call it the “UK NJR”, to account for this we have changed all references to say “UK based National Joint Registry”.

***2. Were the 5100 POPFF identified as having been treated with revision surgery or internal fixation those identified in the NJR only?***

These fractures were identified using a mixture of NJR and hospital episode coding data. The NJR only identifies revision operations until very recently. The data we used was Primary surgery with linked revision operations only.

***Were the 2831 fractures those identified in the NHS data only? Were any fractures identified in both datasets? That is, please clarify that the 2831 fractures are not included in the NJR dataset.***

2831 fractures were treated with fixation alone and so were not seen in the NJR dataset.

*“2,831 of these fractures were treated with fixation alone, meaning 56% were not represented in the NJR”*

***3. Figure 2 - please state which figure corresponds to before and after inclusion of POPFF in the caption.***

Thank you, the figure title has been, updated to clarify this.

*“Figure 2 - Reasons for revision of THR documented by the NJR before (top) and after (bottom) inclusion of post-operative periprosthetic femoral fracture (POPFF) treated with internal fixation identified in this study.”*

***Is the only difference between the top and bottom histogram the addition of the 2831 fractures identified in the NHS data?***

*Yes, this is correct.*

***4. Please include the number of patient-years the 809,832 included THRs represents when describing incidence.***

We have now added it in as you suggest, it had been omitted from the incidence section to avoid repetition from earlier in the results, but it is important, and we are happy to include again.

*“We observed that 22,647 of the 809,832 patients (2.8%) underwent surgery for revision (for any cause) or POPFF fixation for any reason, during a total of 5,542,332 patients years giving an incidence rate of 4.09 reoperations per 1000 patient years.”*

***5. What is PTIR? This acronym appears first just after Figure 3 but is not defined.***

Thank you, the prosthesis time incidence rate (PTIR) is defined in the methods section `Statistical analysis`. In the context of the NJR we tend to use Prosthesis Time Incidence Rate rather than Person Time Incidence Rate as each patient may have more than one prosthesis in place.

*“For patients over the age of 70 at the time of primary THR, overall Prosthesis Time Incidence Rate (PTIR) for revision for any cause or POPFF fixation was 4.09 per 1000 patient years and PTIR for POPFF was 1.31 per 1000 patient years.”*

***6. What is the shaded area on Figure 4? Please include counts of THAs and events at 0, 5, 10, 15 and 20 years below this figure.***

Apologies this was not clear. The figure title has been updated:

` *The shaded area indicates 95% confidence interval of the cumulative incidence estimate.`*

***7. The patient demographics section just after Figure 4 should appear as the first subsection of the Results. The Table of demographics of the overall cohort should appear in the main paper.***

Thank you for this suggestion. We agree that demographics are more suited to be earlier in the results section and we have now moved this. We have also moved the demographics of the overall cohort back into the main manuscript.

***8. Table 1 provides p-values assessing the differences in various demographic factors for patients who developed POPFF or not. I don't think that Table 1 is particularly useful - if interest is in knowing what demographic factors are causally related to the development of a POPFF, then confounding of the relationship between each of these factors and the outcome must be considered in turn, adjusting for other factors to reduce confounding of this relationship (and a causal diagram should be constructed to aid in selection of these factors). I recommend deleting this and all discussion of this Table. (For an in-depth discussion of this issue, I recommend Hernan and Robins' textbook What if?) Also note that when p-values are presented, it is essential to state which statistical tests were used to generate these.***

Thank you for your comments. Table 2 shows the difference between patients with and without POPFF, which we feel is a useful description of the demographics of the population with the disease. Our intention is not to show causation but rather description of a cohort. We feel that this table gives a good context for readers who are looking to compare this work to other similar work in the field or perform metanalysis of observational results.

We agree that p values are not informative, particularly in view of the large sample sizes and these can be removed for clarity.

***9. In Figure 5, include the sample size at each year along with cumulative number of events. Please also state what the pink shading is in the caption.***

Risk table, cumulative events table and explanation of shading has been added.

***10. How many hospital stays in the Length of Stay section were concluded with discharge from hospital, and how many with in-hospital mortality?***

175 (3.5%) patients died as an inpatient and the remaining were discharged from hospital.

***Minor comments:***

***1. Introduction: please change "patient's report a loss" to "patients report a loss"***

Thank you for highlighting this. We have now corrected the mistake.

***2. When it is stated in the Introduction that the NJR is linked to itself, does this mean that records from the same patient are linked to each other?***

Yes, that is correct. A surgeon (or member of their team) will complete a form for any primary or primary and revision operation which is then uploaded to the database. If a revision form is received and matches the NHS number and side of a primary operation, then the database can highlight that a revision has occurred and will inform the surgeon. We have added some text to add clarity.

*“This registry is linked to the Office for National Statistics (for mortality outcomes) and to itself to identify revision operations (if a revision form is submitted, it will be linked to the corresponding primary record if it is held in the NJR);…”*

***3. Please provide a reference for the statement "There are 520 additional revisions… this is roughly in keeping with results of the NJR annual data quality audits."***

Thank you for highlighting this omission. The reference has now been added and is seen below for your reference.

*“Ben-Shlomo Y, Blom A, Boulton C, et al. National Joint Registry Annual Reports. The National Joint Registry 20th Annual Report 2023. London: National Joint Registry*

*© National Joint Registry 2022.; 2023.”*
