# Supplementary material for: The incidence of postoperative periprosthetic femoral fracture following total hip replacement: An analysis of UK National Joint Registry and Hospital Episodes statistics data
Source: PLoS Med. 2024 Oct 1;21(10):e1004462. doi: 10.1371/journal.pmed.1004462 (PMC11444412; doi:10.1371/journal.pmed.1004462)
Supplement: Attachment — Submitted filename: Response_to_reviewers_2.docx [file pmed.1004462.s006.docx]

24^th^ July 2024

Dear Ms Sunny,

**PMEDICINE-D-24-01147R2 - Postoperative periprosthetic femoral fracture is the leading cause of reoperation following total hip replacement: An analysis of linked data from the National Joint Registry and Hospital Episodes Statistics.**

We thank you and the reviewers for your helpful reviews and comments on our manuscript. We have addressed your comments and queries as detailed below and have altered the manuscript accordingly. We would be very grateful for your further review for suitability for publication in PLOS Medicine.

Thank you very much again for your time,

Yours sincerely,

Jonathan T Evans on behalf of the authors

Clinical Senior Lecturer in Trauma & Orthopaedics

University of Exeter, UK

**Editorial comments:**

**We thank you for your thorough revision and we continue to find your research interesting and of clinical importance. As mentioned previously, we invite you to submit a revised manuscript.**

Thank you.

**We ask that you address the academic editor's comments (below) in full; we believe she has highlighted a significant issue. It would be beneficial to state whether the strategy used to identify patients with postoperative periprosthetic femoral fracture (POPFF) was validated and, if not, we ask that you make an acknowledgement of this in your main text.**

Thank you, we have not undertaken a formal validation of the codes used to identify the fractures and as such have included an acknowledgement of this in the main text.

*“A combination of ICD10 and OPCS4 codes in the HES data were used to identify fractures that had undergone surgery, occurred on the same side as the linked primary hip replacement and after the date of the primary (see Table 1 of Supplemental material). This set of codes has not been independently validated for specificity in identifying POPFF. In addition, HES data were used to identify patients that had undergone revision THR and not been reported to the NJR by using the ICD10 and OPCS4 codes already provided by the NJR for use in the annual data quality audit. ”*

**Considering the limitations of the data, we also ask if you would consider re-wording the conclusions asserted in all relevant sections to take into account that the incidence of POPFF as a complication of total hip replacement may not be as significant as stated in this and the original version of the manuscript.**

Thank you, we understand your concerns and have made appropriate changes to the conclusion of both the abstract and the main manuscript to account for this.

**Abstract conclusion**

*“To date, the incidence of POPFF may have been underestimated with over 50% of cases missed if the case identification in this study is correct. After including these cases, we observed that POPFF is the largest reason for major reoperation following THR and patients sustaining these injuries have a high risk of death. The prevention and treatment of POPFF and requires further resource allocation and research.”*

**Main manuscript conclusion**

*“Periprosthetic femoral fracture may be the largest cause for major re-operation following total hip replacement. Revision only data analysis appears to miss over half of patients who sustain this life changing injury if our method of data capture is accurate. POPFF is associated with high mortality and long stays in hospital both before and after surgery. Research is needed to improve care for this under researched patient group.”*

**Finally, there has been some comments about Figure 2 and the lack of inclusion of counts for 'other' causes of revision that might have been missed in the NJR for what appears to be similar reasons as with POPFF. We think that the readers would appreciate reading the counts for this and this might help them to better contextualise POPFF amongst other complications of total hip replacements. We ask that you address this point further in your second revision.**

Thank you. We agree with your editors and reviewers that this is a good suggestion. Rather than extend the figure to make it wider, we have provided a table detailing the breakdown in the supplemental material and have referred to it under the figure.

**Table 2 - Breakdown of reasons for revision including items contained within "Other"**

| **Indication for revision** | **N** |
| --- | --- |
| Dislocation/subluxation | 4060 |
| Infection | 3527 |
| Periprosthetic fracture stem | 3186 |
| Aseptic loosening stem | 2567 |
| Aseptic loosening socket | 2422 |
| Pain | 1433 |
| Other indication for revision | 978 |
| Malalignment socket | 977 |
| Wear of acetabular component | 862 |
| Lysis socket | 538 |
| Malalignment stem | 497 |
| Lysis stem | 472 |
| Adverse soft tissue reaction to particle debris | 385 |
| Implant fracture stem | 351 |
| Periprosthetic fracture socket | 316 |
| Dissociation of liner | 302 |
| Implant fracture socket | 259 |
| Implant fracture head | 101 |
| Incorrect sizing head socket mismatch head | 69 |
| Incorrect sizing head socket mismatch socket | 61 |
| Incorrect sizing MDS2 head socket mismatch | 9 |

**Comments from the academic editor:**

**The academic editor believed that the topic was 'interesting and clinically relevant' (in keeping with the sentiments of the reviewers and editors). However, she was concerned that the incidence of POPFF might be overestimated by the methods you have used here. She asked that the reliability of the combination of codes used to identify POPFF be clarified and the risk of misdiagnosis (with differential diagnoses) using these codes be discussed as a limitation.**

Thank you, as per the comments above, we have now included statements to this effect in the conclusions and methods sections of the manuscript. Following this suggestion we have also added the following text into the limitations.

*“The codes used to identify POPFF in the linked database have not been formally validated as both sensitive and specific to identify only true cases as the pseudonymised data does not allow this. As a result, this may result in over or under identification of cases, the ratio of cases treated with revision and fixation is however consistent with data collected by the NHFD.^7,9^ ”*

**Comments from the reviewers:**

**Reviewer #1: A much improved manuscript with greater clarity.**

Thank you.

**Some simple grammar corrections required.**

We have been through the manuscript and corrected everything we can find. Hopefully any further errors will be picked up by copy editors if this manuscript is accepted.

**Perhaps figure 2 could be simplified by discarding the part 2 and a hatched portion added to the periprosthetic fracture column rather than repeating the whole histogram with only change.**

Thank you for this suggestion. We have created the suggested figure and are happy for the handling editor to make the decision as to the preferred version.

*“Figure 2 shows the most common reasons for revision, classified into the sub-groups used in the NJR annual report but calculated using our study dataset. The grey bars in the figure represent revision operations and the black area represents the additional POPFF treated with internal fixation identified by this study.”*

“Figure 2 - Reasons for revision of THR (grey bars) documented by the NJR with the inclusion of post-operative periprosthetic femoral fracture (POPFF) treated with internal fixation (black area) identified in this study.

Note: A breakdown of the indications for revision grouped within the “Other” category is provided in the supplemental information.”

**Reviewer #3: I thank the authors for their responses to my comments on the previous version of this manuscript. I have no further comments.**
